# Diagnostic performance of fecal *Helicobacter pylori* antigen test in Uganda

**DOI:** 10.1186/s12876-022-02551-z

**Published:** 2022-12-14

**Authors:** Jacob Canwat Owot, Caleb Tuhumwire, Christine Tumuhimbise, Florence Tusiime, Byaruhanga Emmanuel, Boniface A. E. Lumori, Samson Okello

**Affiliations:** 1 Mbarara Regional Referral Hospital, Mbarara, Uganda; 2 Department of Surgery, Mbarara University of Science and Technology, Mbarara, Uganda; 3 Department of Internal Medicine, Mbarara University of Science and Technology, P. O. Box 1410, Mbarara, Uganda; 4 Department of Population and Global Health, Harvard TH Chan School of Public Health, Boston, USA; 5 Department of Epidemiology, Gillings School of Global Public Health, University of North Carolina at Chapel Hill, Chapel Hill, USA

**Keywords:** Fecal *H. pylori* antigen test, 16S rRNA, Diagnostic test, Uganda

## Abstract

We evaluated the diagnostic performance of a qualitative stool antigen test (SAT) in individuals with dyspepsia in rural Uganda using the polymerase chain reaction-based 16S ribosomal RNA assay (16S rRNA) for nucleotide sequences for two common *H. pylori*-associated genes as the reference standard. We enrolled 150 adults with dyspepsia with no self-reported use of antibiotic and/or antiacid medication medications within a fortnight. We performed blinded SAT on fecal specimens and 16S rRNA tests on gastric specimens. Using nonlinear mixed models, SAT had a sensitivity of 85⋅1% (95%CI 76⋅54%, 93⋅6%), and specificity of 97⋅6% (95%CI 94⋅3, 100). Twelve individuals with dyspepsia need to be tested to correctly diagnose 10 with *H. pylori* infection using SAT. The SAT is a robust diagnostic test to improve the diagnosis of *H. pylori* infection in people with dyspepsia in resource-limited settings.

**Supplementary Information:**

The online version contains supplementary material available at 10.1186/s12876-022-02551-z.

## Background

Over 50% of people in developing countries have their *Helicobacter pylori* (*H. pylori*) infection partly due to the ubiquity of factors such as lack of clean water supply, poor sanitation, and overcrowding that are conducive to oral-fecal disease transmission [1]. *H. pylori* is a class I carcinogen [2] for gastric cancer which ranks as the second leading cause of cancer-related deaths and the fourth most common cancer worldwide [3].

Though the risk of gastric cancer is unknown in developing countries, for Japanese men (a low prevalence group), *H. pylori* infection confers up to 17% lifetime risk of gastric cancer (compared to 1% for uninfected) [4]. Despite the availability of *H. pylori* eradication therapy, host genetics, environmental factors, and bacterial virulence limit the effectiveness of therapy [5]. *H. pylori* strains that disproportionately express the Vacuolating cytotoxin (*VacAs1*) and Cytotoxin-associated antigen A (*CagA*) genes predispose to severe disease [6, 7]. Moreover, the ability to type the infecting *H. pylori* strain to guide clinical care in endemic geographical regions is limited thus more virulent strains accrue from prolonged exposure to ineffective therapy.

Polymerase chain reaction (PCR) based methods for the detection of *H. pylori* have high sensitivity (> 95%) and specificity (˃95%) [8] and provide additional evidence of antibiotic-use-induced coccoid forms that other *H. pylori* detection methods do not [9, 10]. Amplification of at least two or more target genes increases the specificity of *H. pylori* diagnosis and reduces the false-positive rates [11]. Insofar as *H. pylori* antigen tests that utilize fecal specimens have been available for decades, the diagnostic performance of this easy-to-use and the non-invasive test has not been evaluated in *H. pylori* endemic settings.

A robust and cheap test is important for diagnostic programs for populations at the highest risk of *H. pylori* infection and may improve early diagnosis thereby reducing gastric cancer incidence, prevalence, and mortality. We aimed to estimate the diagnostic performance characteristics of a point-of-care qualitative stool antigen test using the presence/absence of nucleotide sequences of two *H. pylori* virulent genes as the reference standard in southwestern Uganda.

## Methods

We conducted a diagnostic study between March 2018 and April 2019 at Mbarara Regional Referral Hospital (MRRH) in southwestern Uganda. We consecutively enrolled adults (aged 18 years or greater) referred to the endoscopy unit for first-ever esophagogastroduodenoscopy (EGD). Those screened for enrollment had to have dyspepsia as an indication of EGD and no prior clinical diagnosis of any upper gastrointestinal cancer. The following sequential exclusions were applied; symptoms of gastrointestinal bleeding within seven days, and self-report use of any antibiotics, H_2_-receptor antagonists, proton-pump inhibitors, or non-steroidal anti-inflammatory (NSAIDs) medications within a fortnight before referral.

### Socio-demographic data

Before EGD and after obtaining informed consent, a trained research assistant administered a standardized questionnaire to capture socio-demographic data including age and sex. Participants were asked to provide a pea-sized fecal specimen which was tested for the absence/presence of *H. pylori* antigen.

### Helicobacter pylori antigen test

A laboratory technician blinded to participant clinical data performed *H. pylori* antigen testing for all fecal specimens following standardized procedures. Briefly, about 50 mg of the fecal specimen was added into a sample collection tube containing 1 ml of assay diluent [Phosphate buffer (20 mM), Bovine serum albumin (1%), Sodium azide (0.01%), Sodium chloride (0.1 M), Tween 20 (0.1%)] to make a fecal solution. Three drops (about 80 μl) of the fecal solution were later added to the sample well of the SD Bioline *H. pylori* antigen testing kit (STANDARD DIAGNOSTIC, INC. Giheung-gu, Korea). This test kit contains the mouse monoclonal anti-*H. pylori* antibodies. After 10 to 15 min, the test kit was inspected for the intensification of the control and test lines. A positive result is when both the control line and test line are intensified.

### Collection of gastric tissue

Following a standardized protocol for routine diagnostic esophagogastroduodenoscopy (EGD), we collected a pair of fundal, corpus, and antrum biopsy specimens from each participant. The gastric tissue was immediately placed into a vacutainer tube with *H. pylori* transport medium before being sent to the laboratory (within 1 h) for storage in a − 80 °C freezer.

### Polymerase Chain Reaction assay for 16S ribosomal RNA in gastric tissue

After extraction of DNA from gastric tissue specimen following standard procedure (Supplementary), a separate laboratory technician blinded to participant clinical data and *H. pylori* stool antigen test results performed PCR amplification of *VacAs1* gene and *CagA* gene nucleotide sequences in gastric tissue using forward *H. pylori* primers:16S rRNA-F: 5’-GCGCAATCAGCGTCAGGTAATG-3’ and reverse *H. pylori* primers:16S rRNA-R: 5’-GCTAAGAGAGCAGCCTAT GTCC-3’ targeting a 503 bp PCR product [12]. The PCR reaction was performed in a 12.5 μl volume containing, 6.25 μl 2.0X TaqMix, 0.25 μl (10 pmol) of each primer, 3.75 μl of nuclease-free water, and 2.0 μl of DNA template. The mixture was subjected to an initial denaturation at 94 ºC for 10 min, followed by 34 cycles of denaturation at 94 ºC for 30 s, annealing at 55 ºC for 60 s, and extension at 72 °C for 60 s, and final extension at 72 °C for 10 min. Amplification was performed in an S1000 ™ Thermal Cycler (BIO-RAD, California, United States). PCR products were electrophoresed in a 2% ethidium bromide-stained agarose gel.

PCR amplification was performed for *VacAs1* and *CagA* gene nucleotide sequences separately. Later, BLAST was used to confirm PCR-amplified 16S rRNA *VacAs1* and *CagA* genes nucleotide sequences. Of note, sequence analysis performed on both *VacAs1* and *CagA* gene nucleotide sequences amplified from *H. pylori* DNA showed a high level (100%) of identity between *H. pylori* strains of the current study with other sequences in the Genbank (Supplementary). We characterized specimens with *VacAs1* and *CagA* gene nucleotide sequences as positive for 16S rRNA and those without were defined as 16S rRNA test negative.

### Statistical analysis

With the 16S rRNA test as the reference standard comparator to the SAT (index test), we fit a saturated nonlinear mixed model with Poisson distribution and log link function without intercept (equation below) for all participants and stratified by sex separately.$\lambda = {e}^{{\beta }_{1}{X}_{1}+{\beta }_{2}{X}_{2}+{\beta }_{3}{X}_{3}+{\beta }_{4}{X}_{4}}$

Where *λ* is the number of participants with positive sRNA in the study sample.

*e*^(β^_1-4_^)^ is the number of participants with positive sRNA for each change in X.

*Χ*_*1*_ is the number of participants who tested negative for both SAT and 16S rRNA tests.

*Χ*_*2*_ is the number of participants with negative SAT and positive 16S rRNA results.

*Χ*_*3*_ is the number of participants with positive SAT and negative 16S rRNA results.

*Χ*_*4*_ is the number of participants who tested positive for SAT and 16S rRNA results*.*

We obtained estimates of the diagnostic performance characteristics for SAT, including *i)* sensitivity (the proportion of participants with positive 16S rRNA test who have a positive SAT), *ii)* specificity (the proportion of participants with negative 16S rRNA test who have a negative SAT), *iii)* the positive predictive value (the probability of a positive 16S rRNA test if a participant has a positive SAT), *iii)* negative predictive value (the probability of a negative 16S rRNA test if a participant has a negative SAT), *iv)* the likelihood ratio of a positive test (how much more likely is a positive SAT is in a participant with a positive 16S rRNA test than in a participant with a negative 16S rRNA test), *v)* the likelihood ratio of a negative test (how much more likely is a negative SAT is in a participant with a positive 16S rRNA test than in a participant with a negative 16S rRNA test), *vi)* diagnostic accuracy (how much more likely the SAT would make a correct diagnosis than an incorrect diagnosis in participants with positive 16S rRNA test), and *vii)* the number needed to diagnose (NND) (the number of participants that have to be tested for SAT to give one correct diagnosis). The standard errors were obtained using the Delta method [13].

In addition, we estimated the diagnostic performance of the SAT in other populations with varying *H. pylori* prevalence between 15 and 90% to describe the diagnostic performance of SAT in those settings. All analyses were performed using SAS 9.4 (Cary, NC, USA).

## Results

After exclusions, data from 150 participants with dyspepsia were analyzed. The mean age was 55 (standard deviation 16⋅8) years, age ranged from 19 to 90 years, and 78 (52%) were females. Fifty-nine (39⋅3%) tested positive for the stool antigen test. Of the 67 (44⋅7%) participants categorized as positive for 16S rRNA (i.e., with nucleotide sequences of *H. pylori*-associated genes in gastric tissue), 22 (32⋅8%) had the *VacAs1* gene nucleotide sequences, 14 (20⋅9%) had the *CagA* nucleotide sequences, and 31 (46⋅3%) expressed both *VacAs1* and *CagA* gene nucleotide sequences.

Fifty-seven were positive for SAT and 16 sRNA nucleotide sequences thus the sensitivity of 0⋅85 (95%CI 0⋅76, 0⋅94) and positive predictive value of 0⋅97 (95%CI 0⋅92, 1⋅01). Men had higher sensitivity and positive predictive value than women. Seventy-nine were negative for SAT and 16S rRNA nucleotide sequences thus the specificity of 0⋅98 (95%CI 0⋅94, 1⋅01) and the negative predictive value of 0⋅89 (95%CI 0⋅83, 0⋅95). The specificity and negative predictive value were similar for men and women (Table 1). About 12 individuals with dyspepsia need to be tested with SAT to correctly diagnose 10 individuals as would be positive with 16S rRNA.

**Table 1.** Diagnostic performance of H. pylori stool antigen test in southwestern Uganda

	**16s rRNA**	**Fecal antigen**	**n (%)**	**sensitivity**	**specificity**	**PPV**	**NPV**	**LR-**	**LR+**	**NND**
Men, n=72	Positive	Positive	26 (36.11)	0.81 (0.62, 1.00)	0.97 (0.91, 1.04)	0.96 (0.86, 1.06)	0.87 (0.73, 1.01)	0.19 (-0.01, 0.39)	32 (-57, 122)	1.21 (0.95, 1.46)
		Negative	6 (8.33)							
	Negative	Positive	1 (1.39)							
		Negative	39 (54.17)							
Women, n=78	Positive	Positive	31 (39.74)	0.89 (0.74,1.03)	0.98 (0.91, 1.04)	0.97 (0.88,1.05)	0.91 (0.80,1.03)	0.12 (-0.04, 0.27)	38 (-67,143)	1.13 (0.95, 1.32)
		Negative	4 (5.13)							
	Negative	Positive	1 (1.28)							
		Negative	42 (53.85)							
All, n=150	Positive	Positive	57 (38)	0.85 (0.76, 0.94)	0.96 (0.94, 1.01)	0.97 (0.92, 1.01)	0.89 (0.83, 0.95)	0.15 (0.06, 0.24)	35 (-13, 84)	1.17 (1.06, 1.28)
		Negative	10 (6.7)							
	Negative	Positive	2 (1.3)							
		Negative	81 (54)							

*PPV* Positive Predictive Value, *NPV* Negative Predictive Value, *LR-* Likelihood Ratio of a negative test, *LR+ *Likelihood Ratio of a positive test, *NND* Number Needed to Diagnose. H. pylori stool antigen test accuracy for overall sample is 0.92 (95%CI 0.88, 0.96); for women only is 0.94 (95%CI 0.86, 1.01); and men only is 0.90 (95%CI 0.81, 0.99). F1 score for overall sample is 0.90 (95%CI 0.85, 0.96); for women only is 0.92 (95%CI 0.83, 1.02); and men only is 0.88 (95% CI 0.76, 1.00)

**Fig. 1:**
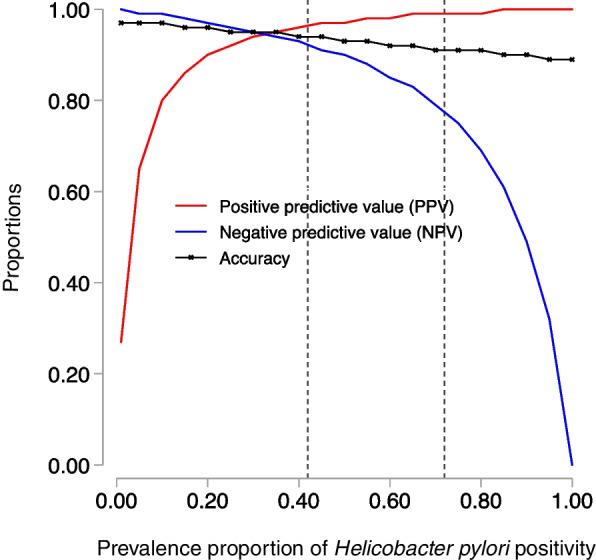
Variability of positive predictive value (PPV), negative predictive value (NPPV), and accuracy of the *Helicobacter pylori* stool antigen test by the prevalence of *Helicobacter pylori.* Footnote: The vertical dotted lines are the lowest reported prevalence of *Helicobacter pylori* in Uganda (42% in the community) and highest (72% among patients with dyspepsia in a hospital setting)

As the prevalence of *H. pylori* increased towards 100% with virtually no false negatives on the SAT (near-perfect sensitivity), the probability of a negative 16S rRNA test associated with a negative SAT (the negative predictive value) decreased. Conversely, as the prevalence decreased toward 0%, even if the SAT had near-perfect specificity (close to 100%) with virtually no false positives, the probability of a positive 16S rRNA test associated with a positive SAT (the positive predictive value) decreased. Taken together, the SAT results are most ‘predictive’ when *H. pylori* prevalence is at 30% (Fig. 1). Based on the estimated specificity and sensitivity, given a 72% prevalence of *H. pylori* among Ugandans presenting to a hospital with dyspepsia [14], for a hypothetical population of 100 Ugandans, 66 would test positive by SAT and 16S rRNA (specificity of 0.89 and specificity of 0.98). Among those who will test positive, 99% will be positive by 16S rRNA, and 75.5% of those who will test negative will not have *H. pylori* by 16S rRNA (Fig. 1).

## Discussion

In the current study evaluating the diagnostic performance of a point-of-care qualitative stool antigen test in people with dyspepsia in rural southwestern Uganda, we found that the SAT had excellent diagnostic performance characteristics for the diagnosis of *H. pylori* in this disease-endemic setting. Furthermore, we found that twelve individuals with dyspepsia need to be tested with SAT to correctly detect ten individuals with *H. pylori* infection among people with dyspepsia.

Studies comparing SAT to immunohistological *H. pylori* testing in low-income settings show a low sensitivity [15, 16] perhaps because most individuals tested had bleeding peptic ulcers, a known cause of false-positive *H. pylori* [17]. However, using similar comparison methods of SAT with immunohistology, Erzin et al. [18] found high sensitivity and low specificity compared to this current study. Our study used the presence/absence of PCR-16S rRNA to define positive/negative *H. pylori* as a reference standard, whereas their study used a more stringent definition for positive *H. pylori* of culture alone or both the histology and the rapid urease test [19]. Our findings are consistent with those from a meta-analysis of eighty-nine studies, including 10,858 untreated adults in Europe and Asia, which found similar diagnostic performance characteristics of monoclonal stool antigen tests in pretreatment individuals [20]. However, the studies included used standard tests with lower sensitivities and specificities like rapid urease test, culture, urea breath test, IgG-antibody-based serology, histology, and immunohistostaining bar PCR-based tests [20]. In addition, the prevalence of *H. pylori* infection in the geographical settings of the studies included is lower than in individuals with dyspepsia in Uganda.

Though the specificity and negative predictive value in this current study were similar in both sex, males had higher sensitivity and positive predictive value than females. Similarly, Krausse et al.found a higher performance in SAT in males [21]. On the contrary, Abdelmalek et al.found no difference in the diagnostic performance of SAT in Egypt [22]. These findings may reflect potential differences in exposure mechanisms by gender roles.

This current study found the prevalence of *H. pylori* infection at 39.3% and 44.7% using SAT and PCR-16S rRNA, respectively. These are in keeping with other hospital-based studies in the region that found *H. pylori* prevalence by SAT of 33.5 – 35.7% and 32.5% by immunohistology [15].

The strength of the current study is the blinding of laboratory technicians who performed SAT and PCR to the clinical diagnosis of participants reducing the differential misclassification bias that would affect the estimates. However, the findings herewith may be extrapolated to other settings with high *H. pylori* burden with caution that; first, this was a single-hospital study with relatively small sample size. Second, 16S rRNA PCR analysis was done on genomic DNA isolated from gastric tissues which may have underestimated the *VacAs1* and *CagA* prevalence in the study population due to potential PCR inhibitors.

In conclusion, the stool antigen test is a non-invasive and robust diagnostic test that may be used to improve the diagnosis of *H. pylori* infection in people with dyspepsia in *H. pylori* endemic resource-limited settings.

## Supplementary Information

**Additional file 1.**

## Data Availability

The datasets used and/or analyzed in the current study are available from the corresponding author on reasonable request.
