# Supplementary material for: Diagnostic performance of fecal Helicobacter pylori antigen test in Uganda
Source: BMC Gastroenterol. 2022 Dec 14;22:518. doi: 10.1186/s12876-022-02551-z (PMC9749204; doi:10.1186/s12876-022-02551-z)
Supplement: Supplementary file 1 — Additional file 1. [file 12876_2022_2551_MOESM1_ESM.docx]

### Supplement Text

### Extraction of genomic DNA from gastric biopsies

All gastric biopsy tissue specimens were kept at -80˚C freezer after collection by EGD. To extract DNA extraction from the tissues, we added 500µl of STE buffer and water-bathed at 65˚C, incubated for 2 hours, and agitating after every 30 minutes. This was followed by adding 500µl of 24:1 chloroform: Isoamyl alcohol to the tissue specimen and vortex. Phase separation was performed by centrifuging the samples at 13000 rpm for 5 minutes and 400 µl of the supernatant was removed and placed in a new Eppendorf tube (1.5ml). Then 1200µl of ice-cold absolute ethanol and 40µl of 5M Sodium acetate (PH 5.2) were added. Mixing was done by vortexing. This was followed by incubation at -20˚C for 2 hours. After which samples were centrifuged at 1300rpm at 4˚C for 30 minutes.

The supernatant was carefully removed and to it, 70% Ethanol was added, vortex, and centrifuged at 1300 rpm for 30 minutes at 4˚C. The supernatant was removed, and the pellet was air-dried at room temperature for 20 minutes. Finally, 60µl of elution buffer (Tris–EDTA) was added to the pellet and left to dissolve by heating at 65˚C while agitating (1000 rpm) and DNA was stored at -20°C.

### PCR amplification of VacAs1 gene

To amplify the VacA gene, the forward VA1-F: 5’- ATG GAA ATA CAA CAA ACA CAC-3’ and reverse VA1-R 5’- CTG CTT GAA TGC GCC AAA C -3’ primers were used (Atherton *et al*., 1995). The pair of primers amplifies a PCR product of 259 bp. The PCR reaction was performed in a 12.5µl volume containing, 6.25µl (2.0X) TaqMix, 0.25µl (10pmol) of each primer, 3.75µl of nuclease-free water, and 2.0µl of DNA template. The mixture was subjected to an initial denaturation at 94ºC for 10 min, followed by 34 cycles of denaturation at 94 ºC for 30 seconds, annealing at 55ºC for 60 seconds, and extension at 72°C for 60 seconds, and final extension at 72°C for 10 min. Amplification was performed in an S1000 ™ Thermal Cycler (BIO-RAD, California, United States).

### PCR amplification of CagA gene

For amplification of the CagA gene, a pair of primer: CagA –Forward: 5’- GATAACAGGCAA GCTTTTGAG- 3’ and CagA–Reverse: 5’- CTGCAAAAGATTGTTTGG CAG- 3’ targeting a 351bp product was used (Chattopadhyay *et al*., 2004). The PCR reaction was performed in a 12.5µl volume containing, 6.25µl (2.0X) TaqMix, 0.25µl (10pmol) of each primer, 3.75µl of nuclease-free water, and 2.0µl of DNA template. PCR conditions were maintained except that an annealing temperature of 53ºC for 60 seconds was used. PCR products were revealed by 2% agarose gel electrophoresis.
